# Activation of M1 macrophages in sepsis-induced acute kidney injury in response to heparin-binding protein

**DOI:** 10.1371/journal.pone.0196423

**Published:** 2018-05-03

**Authors:** Li Xing, Lu Zhongqian, Song Chunmei, Chen Pingfa, He Lei, Jin Qin, Mu Genhua, Deng Yijun

**Affiliations:** 1 Department of ICU, Yancheng City No.1 People’s Hospital, Yancheng, China; 2 Nursing College of Nantong University, Nantong, China; National Institutes of Health, UNITED STATES

## Abstract

**Background:**

In the early stage of sepsis, M1 macrophages result in the production of inflammatory mediators and AKI. Heparin-binding protein (HBP) have been shown to play important roles in sepsis-induced AKI. In this study, we investigate the association of HBP with M1 macrophages in sepsis-induced AKI.

**Methods:**

Male C57BL6 mice were subjected to cecal ligation and puncture (CLP) or sham surgery. Biochemical and histological renal damage was assessed. Macrophage infiltration was assessed by immunohistochemistry. RT-PCR was used to investigate the expression of heparin-binding protein (HBP), the inducible nitric oxide synthase (iNOS) and arginase 1 (Arg-1) mRNAs. Western blots were performed to assay the tissue levels of HBP, tumor necrosis factor alpha (TNF-α) and interleukin-6 (IL-6).

**Results:**

High levels of HBP were obviously detected 24 h after sepsis-induced AKI. Heparin inhibited HBP expression during sepsis-induced AKI. The suppression of HBP expression by heparin injection after the establishment of sepsis-induced AKI resulted in a reduction in renal injury severity accompanied with a significant repression of M1 macrophage activation and expression of TNF-α and IL-6.

**Conclusions:**

HBP plays an important role in the initial inflammatory reaction associated with sepsis-induced AKI, presumably by activating M1 macrophages and suppressing TNF-α and IL-6 secretion.

## Introduction

Acute kidney injury (AKI) is a common complication in critically ill patients and is associated with increased morbidity and mortality [1]. Sepsis is the most common cause of AKI [2]. Animal models of sepsis have been developed and demonstrate that the pathogenesis of AKI is caused by inflammatory cell infiltration, renal endothelial cell dysfunction, intrarenal hemodynamic alterations, and renal cell apoptosis in the kidney [3]. Sepsis-induced AKI is caused by a combination of multiple mechanisms, including inadequate vascular leakage/perfusion, local tubular inflammation and cell cycle arrest [4]. Of these factors, significant tissue inflammation in the kidney appears to be a critical mediator of sepsis-induced AKI [5]. Recent studies have indicated that innate immunity and inflammatory signaling pathways are involved in the pathogenesis of septic AKI, and these processes are initiated a few hours after injury by the infiltration of immune cells from the kidney and are of central importance to kidney regeneration [6,7].

Macrophages, the most common type of leukocytes involved in the process of renal injury, play different roles at different stages of injury [8]. Because of differences in the immune microenvironment, macrophages can be classified into several phenotypes and functional subclasses that exhibit different functions. In the early stage of sepsis, macrophages undergo M1 differentiation, resulting in the production of inflammatory mediators and AKI [9]. M1 macrophages upregulate the expression of pro-inflammatory mediators, including inducible nitric oxide synthase (iNOS) and tumor necrosis factor-alpha (TNF-α), and increase the production of reactive oxygen and nitrogen species [10]. In contrast, anti-inflammatory M2 macrophages upregulate the expression of arginase-1 (Arg-1), scavenger and mannose receptors, and the intracellular protein found in inflammatory zone 1 (FIZZ1). iNOS expression has been used as a marker of M1 responses, whereas Arg-1 and FIZZ1 are classical inducers of M2 gene expression [11,12].

Heparin-binding protein (HBP), also known as CAP37, is a promising biomarker for predicting the development and prognosis of severe sepsis and septic shock and has recently been proposed to be involved in the pathophysiology of AKI [13,14]. HBP acts as a chemoattractant for neutrophils, T cells and monocytes and enhances monocyte cytokine release, phagocytosis and adhesion to the endothelium [15]. HBP induces inflammation and capillary leakage in the kidney, as demonstrated by the findings of Fisher et al [16]. However, whether HBP induces macrophages to promote inflammation is unknown. To date, there have been no studies on the association of HBP with M1 macrophages in sepsis-induced AKI.

To further improve our understanding of inflammatory processes associated with sepsis-induced AKI mouse, we used a sepsis-induced AKI mouse model to investigate the in vivo expression of HBP. Meanwhile, the suppression of HBP expression by heparin injection after the establishment of sepsis-induced AKI resulted in a reduction in renal injury severity accompanied with a significant repression of M1 macrophage activation and expression of pro-inflammatory cytokines.

## Materials and methods

### Ethics statement

Six- to eight-week-old wild-type male C57BL6 mice were housed in specific pathogen-free cages at 23±2°C and 60±10% humidity, with a 12-hour light/12-hour dark cycle and free access to food and water. All animal procedures were performed in compliance with the Institute of Laboratory Animal Research Guide for the Care and Use of Laboratory Animals of the National Institutes of Health and were approved by the Institutional Animal Care and Use Committee of Nanjing Medical University.

### Septic animal models

CLP was performed using a previously described method [10], with slight modifications. Briefly, a 4–0 silk ligature was placed 15 mm from the cecal tip after laparotomy under isoflurane anesthesia. The cecum was punctured twice with an 18-gauge needle and gently squeezed to express a small amount of fecal material before being returned to the central abdominal cavity. In sham-operated animals, the cecum was located but neither ligated nor punctured. The abdominal incision was closed in two layers with 6–0 nylon sutures. After surgery, animals were fluid resuscitated with 40 ml/kg of subcutaneously administered sterile saline and given free access to water but not food. During the surgical procedure, body temperature was maintained at approximately 37°C. Animals were sacrificed by cervical dislocation after 72 h, blood was collected, and kidney samples were harvested and stored at -80°C until further analysis.

### Unfractionated heparin (UFH) preparation and administration

Unfractionated heparin (UFH) and phosphate-buffered saline liposomes were prepared according to previously described methods [15]. mices received an intraperitoneal bolus of 0.4 U/g body weight of UFH or phosphate-buffered saline liposomes (Formumax, USA) 12 h prior to sepsis-induced AKI, as previously reported.

### Blood chemistry assay

After blood collection, the concentrations of blood urea nitrogen (BUN) and serum creatinine (Scr) were immediately analyzed using a Roche Diagnostic analyzer (Roche, Indianapolis, IN). The Scr content was determined using a creatinine (serum) colorimetric assay kit (Cayman Chem, Ann Arbor, MI).

### Renal histology analysis

Tissues were fixed with 10% formalin and embedded in paraffin. Four-micrometer sections were stained with periodic acid-Schiff (PAS) reagent. Histological changes in the cortex and the outer stripe of the outer medulla (OSOM) were assessed by quantitative measurements of tissue damage. As tubular damage was mainly vacuolization, the damage was defined as tubular vacuolar degeneration. The degree of kidney damage was estimated in 200X magnification images of more than 100 randomly selected tubules for each animal using the following criteria: 0, normal; 1, area of damage <25% of tubules; 2, damage in 25% to 50% of tubules; 3, damage in 50% to 75% of tubules; and 4, 75% to 100% of tubules were affected. The histological analysis was performed by two pathologists graded sections in a blind fashion to avoid bias.

### RT-PCR

RNA was isolated from snap-frozen kidneys stored at 80°C using standard procedures (RNeasy, QIAGEN). RNA was treated with DNase1 (Invitrogen), and reverse transcription was performed using 0.5 g of total RNA (iScript, Bio-Rad). PCR was performed on 1/20 of the RT product using the following primer pairs: iNOS forward 5’-GAATTCCCAGCTCATCCGGT-3’ and reverse, 5’- GGTGCCCATGTACCAAC CGGT-3’; Arg-1 forward, 5’-CCGCAGCATTAAGGAAAGC-3’ and reverse, 5’-CCC GTGGTCTCTCACATTG-3’; HBP forward, 5’-ACAACCTCA ACGTCATCCTG G-3’ and reverse, 5’- GTCTTCATTGAGGGCGTTGC-3’; and β-actin forward, 5’-CAGTAACAGTCCGCCTAGAA-3’ and reverse, 5’-GATTACTG CTCTGGCT CCTA-3’. The cycling conditions were a melting temperature (Tm) of 55°C for 30 cycles, and PCR products were visualized with ethidium bromide staining after separation on 2% agarose gels and photographed.

### Immunohistochemistry

Renal tissue sections were subjected to immunohistochemical staining for F4/80 and Fi67. For immunohistochemical staining, 3-mm renal sections were deparaffinized and rehydrated in a graded alcohol series. The sections were immersed in 3% hydrogen peroxide for 10 min to block endogenous peroxidase activity and then incubated in buffered normal horse serum to block non-specific binding. Prior to immunochemistry, sections were subjected to antigen retrieval by immersion in 0.1 mol/L citrate buffer (pH 6.0) for 25 minutes, followed by heating in an electrical pressure cooker for 5 minutes. Sections were incubated with mouse monoclonal anti-CD68 antibody (1:100, Abcam, USA) primary antibodies overnight at 4°C. Control experiments omitted either the primary or secondary antibody. On the next day, sections were incubated with a horseradish peroxidase (HRP)-conjugated goat anti-rabbit or rat anti-mouse secondary antibody (Beijing Zhongshan Biotechnology Co., Beijing, China) for 1 h at room temperature. Then, 3, 3-diaminobenzidine tetrahydrochloride (DAB, Beijing Zhongshan Biotechnology Co., Beijing, China) was applied to the slides to develop a brown color. Counterstaining was performed with hematoxylin, and photomicrographs were captured with an Olympus camera.

### Western blot analysis

Whole-cell extracts were prepared. Protein samples were resolved on 10% SDS-PAGE gels, transferred onto PVDF membranes, and blocked with 5% skim milk. Subsequently, the primary antibody incubation was performed overnight at 4°C, followed by an incubation with an HRP-conjugated secondary antibody conjugated for 1.5 h. Protein bands were detected using an enhanced chemiluminescence (ECL) detection system (Pierce, Rockford, USA).

### Statistical analysis

The results are presented as the means±SEM. The Wilcoxon test was used to analyze non-parametric data. After the normal distribution of the data was assessed with the Kolmogorov-Smirnov test, statistical comparisons between experimental groups were evaluated using Student's t test and one-way ANOVA with SPSS 20.0 software (SPSS Inc., Chicago, IL, USA); a P value <0.05 was considered significant.

## Results

### Expression of HBP in the kidney following sepsis-induced AKI

**Fig 1:**
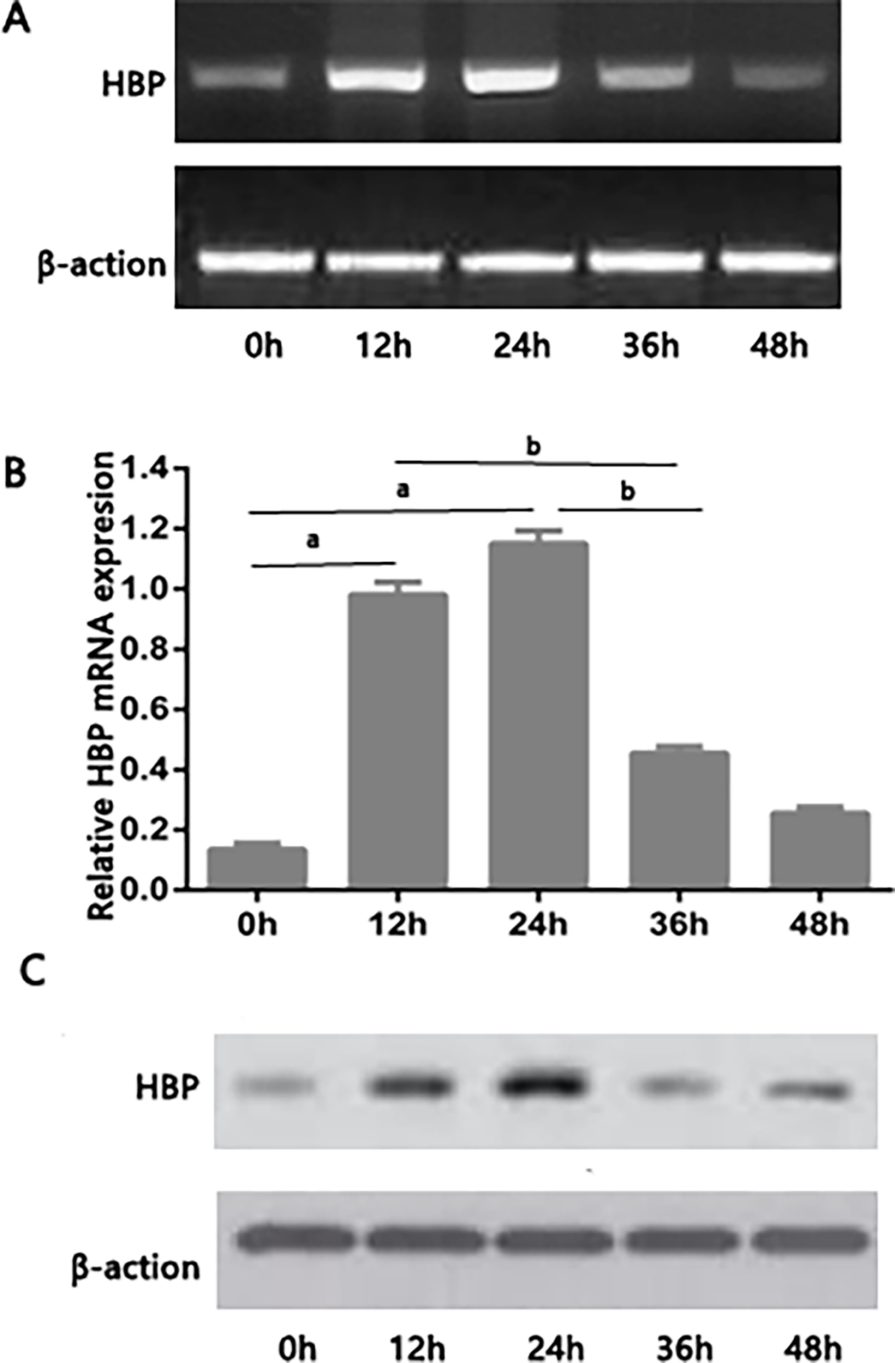
HBP mRNA levels following sepsis-induced AKI in mices. Mices were subjected to CLP surgery. Renal tissue sections were collected from the CLP group at various times (0, 6, 12, 24, 36 and 48h). Levels of the HBP mRNA were measured using RT-PCR (A) and quantified by densitometry (B). The data are presented as the means±standard errors of the means (SEM); ^a^P<0.05 compared with the sham group; ^b^P<0.05 compared with the 36h group (n = 6 mices/group).

To detect the renal HBP production during different sepsis-induced AKI, mice were subjected to cecal ligation puncture (CLP) surgery and sacrificed at various times (Fig 1). Active renal HBP production remained low in sham-operated animals. However, renal HBP production significantly increased at 24 h in response to sepsis-induced AKI and then subsequently decreased. To obtain more detailed evidence, we quantitatively assessed renal HBP expression using real-time PCR analysis. High levels of HBP were detected 24 h post-injury, which decreased over the following days.

### Heparin downregulated HBP expression during sepsis-induced AKI

**Fig 2:**
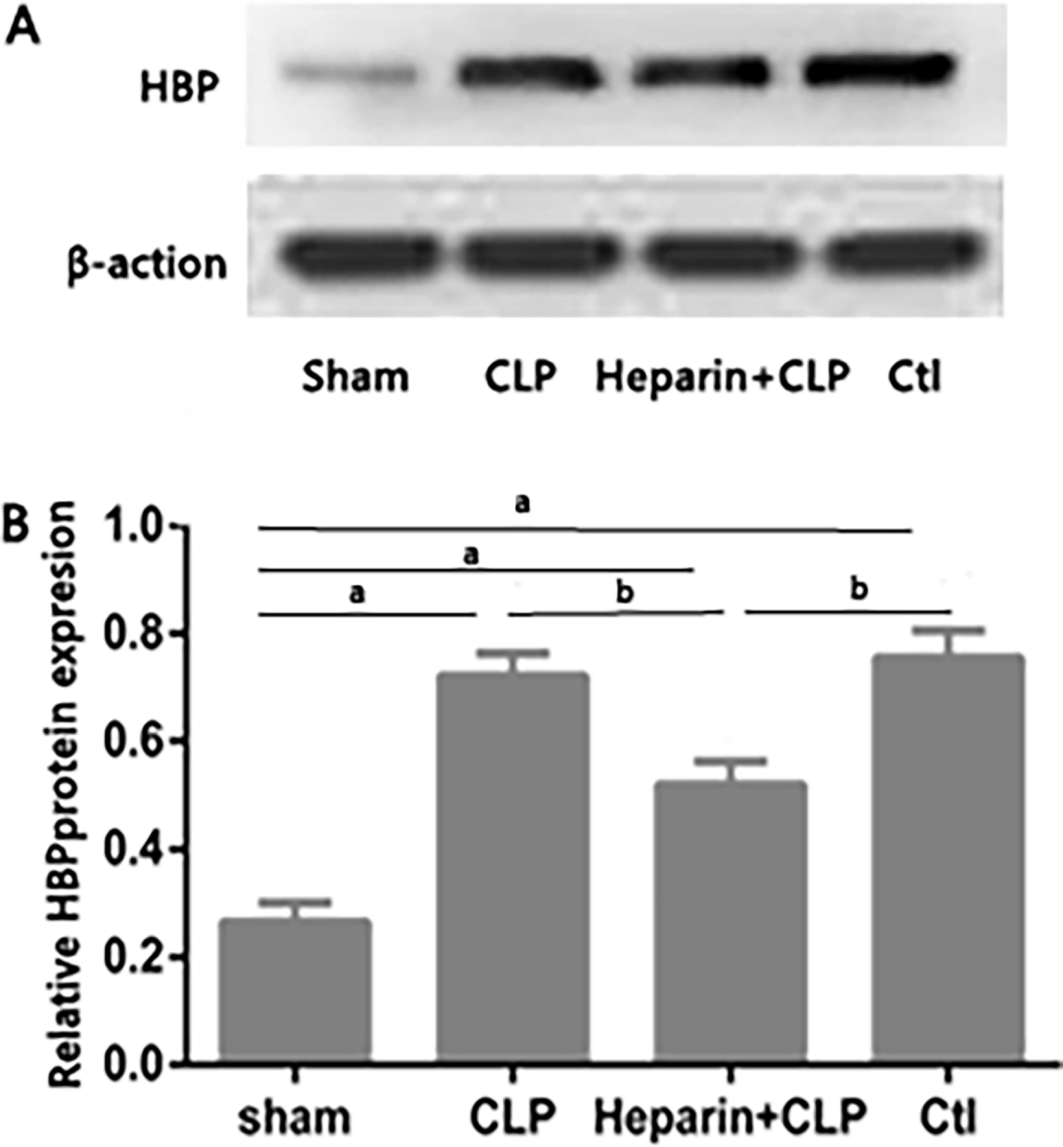
Effect of heparin on HBP protein levels with sepsis-induced AKI in mices. Mices were subjected to CLP surgery. Twenty-four hours prior to the onset of CLP, mices were treated with heparin. Levels of the HBP protein in the kidney were determined by Western blotting (A) and quantified by densitometry (B). Protein levels were normalized to β-actin. Graphs represent means±SEM; ^a^P<0.05 compared with the sham group; ^b^P<0.05 compared with the heparin+CLP group (n = 6 mices/group).

Heparin is a multifunctional negatively charged glycosaminoglycan that binds to HBP. Mice were given an intraperitoneal bolus of heparin 12 h prior to the induction of sepsis-induced AKI. We performed western blotting in mouse renal tissues to determine the expression of HBP at 24 h (Fig 2). The heparin+CLP group exhibited a significant downregulation of TNF-α expression than did the CLP and control (Ctl) groups.

### HBP increased renal damage and dysfunction during sepsis-induced AKI

**Fig 3:**
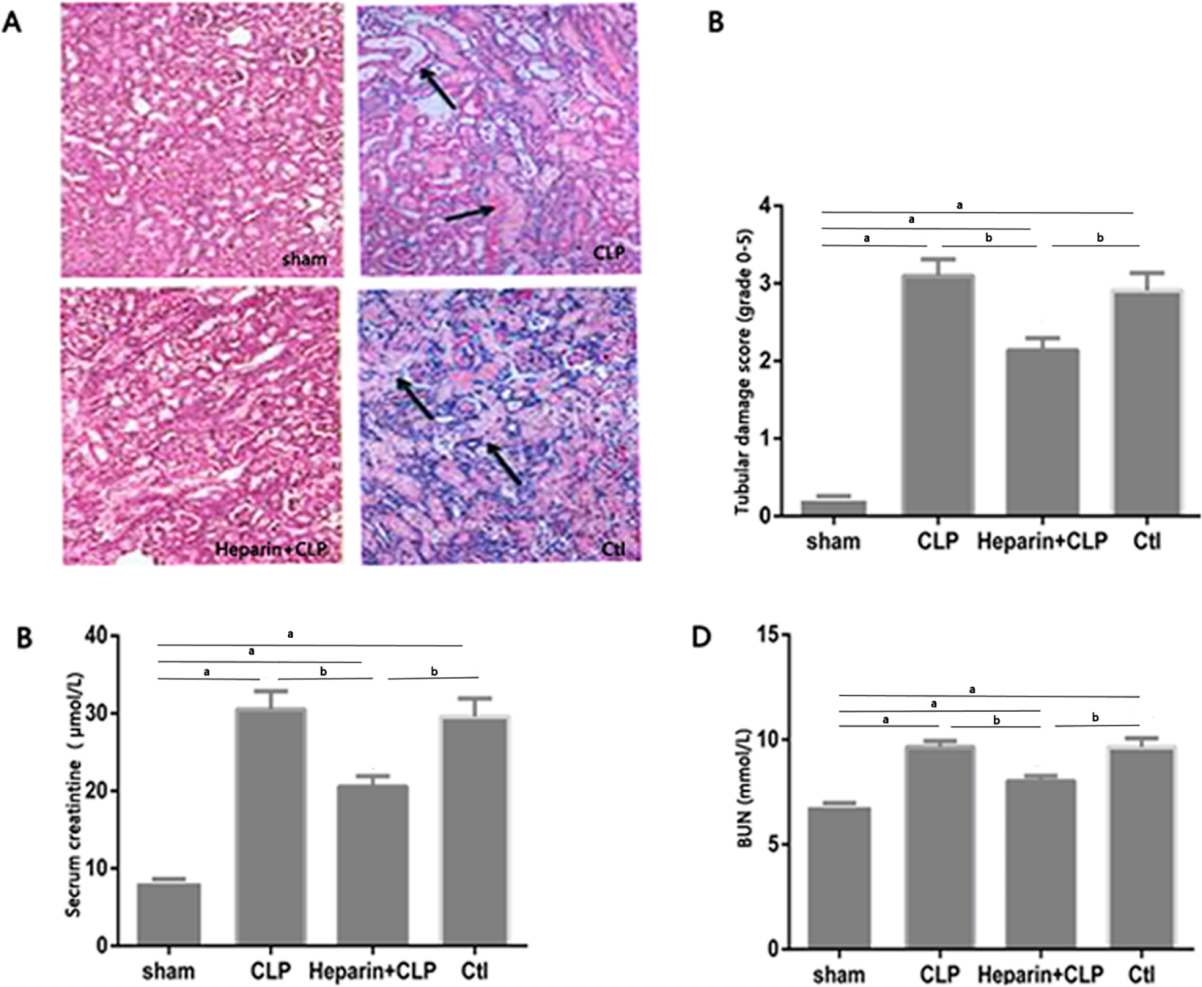
Effect of HBP on histology and renal function in mices with sepsis-induced AKI. (A) Photomicrographs of H&E-stained kidney sections (200x). All fields were chosen from the cortex and outer medulla. (B) Semi-quantitative scoring of histological injury. (C and D) BUN and Scr levels were measured to determine renal function. The data are presented as the means±SEM; ^a^P<0.05 compared with the sham group; ^b^P<0.05 compared with the heparin+CLP group (n = 6 mices/group).

Histological examinations and tubular damage scoring were performed on samples 24 h after sepsis-induced renal injury (Fig 3). Widespread damage was observed in the form of a dilated, flattened, and swollen epithelium and the loss of proximal tubular epithelial cells, along with luminal cast formation. The damage was more severe in the CLP group and the Ctl group than in the heparin+CLP group. The heparin+CLP group exhibited a significant reduction in blood urea nitrogen (BUN) and serum creatinine (Scr) levels than did the CLP and Ctl groups, indicating that HBP increased renal damage and renal dysfunction during sepsis-induced renal injury.

### HBP increased macrophage infiltration during sepsis-induced AKI

**Fig 4:**
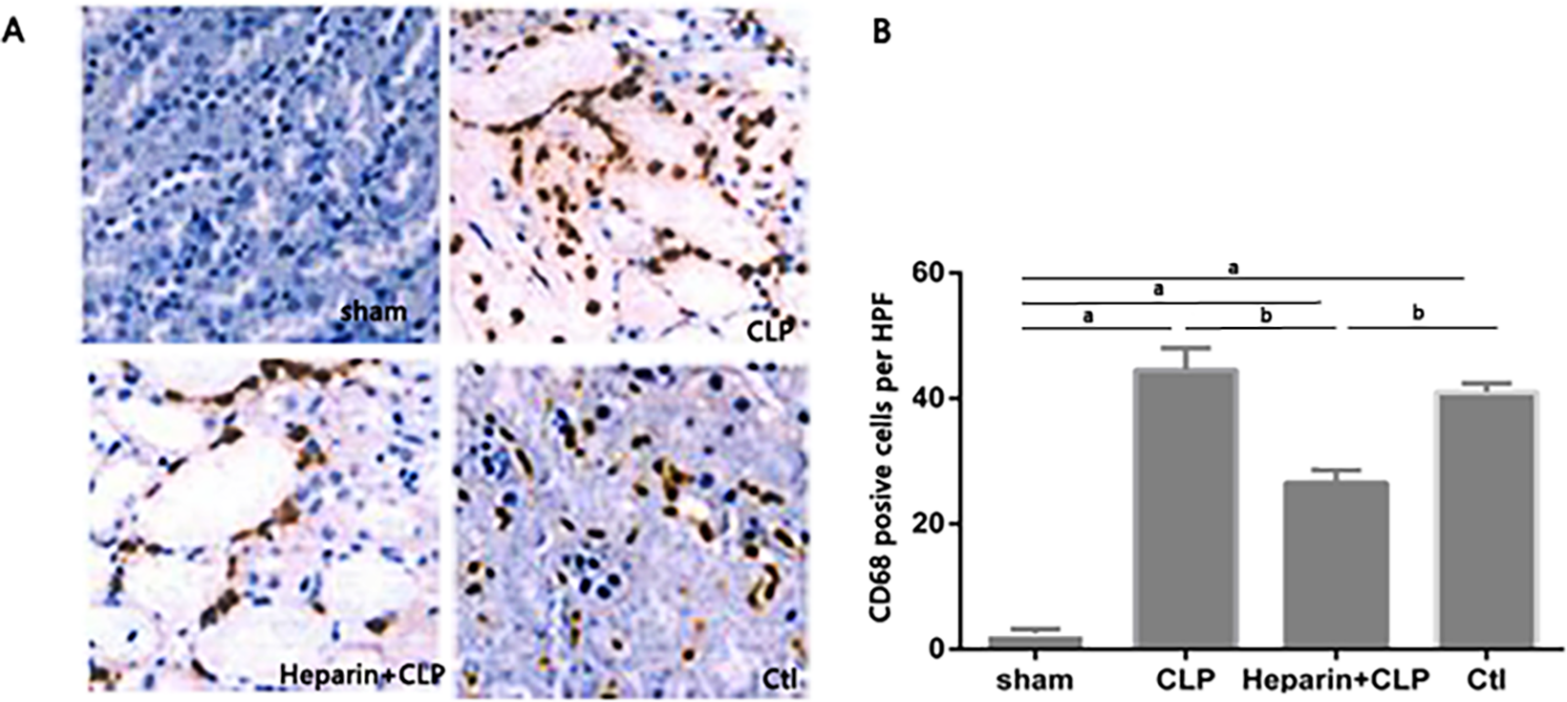
Effect of HBP on macrophage infiltmiceionwith sepsis-induced AKI in mices. (A) Expression of the CD68 protein was detected by immunohistochemical staining (200x). (B)Number of CD68-positive cells per HPF. The data are presented as the means±SEM; ^a^P<0.05 compared with the sham group; ^b^P<0.05 compared with the heparin+CLP group (n = 6 mices/group).

Renal cortical tissue sections were stained with a specific antibodies against the mouse macrophage marker CD68 for the assessment of the degree of macrophage infiltration during renal injury after the CLP procedure (Fig 4). Sham-operated mice exhibited minimal interstitial macrophage staining. However, mice that were subjected to CLP surgery exhibited significant macrophage accumulation after 24 h. The degree of sepsis-induced macrophage infiltration was significantly reduced in the presence of heparin. Thus, HBP induced macrophage activation and infiltration after sepsis-induced AKI.

### HBP promoted M1 differentiation following sepsis-induced AKI

**Fig 5:**
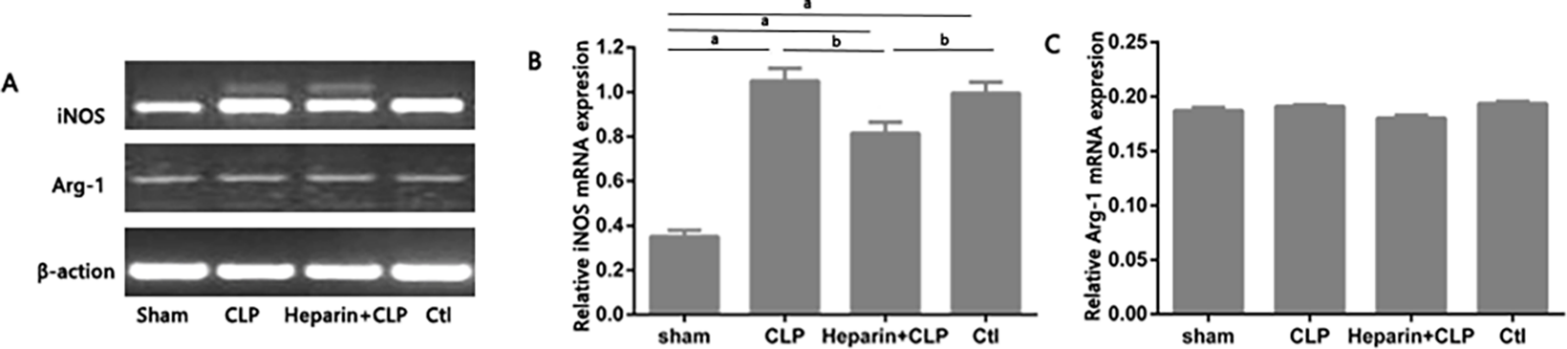
Effect of HBP on M1 macrophage differentiation with sepsis-induced AKI in mices. Levels of the iNOS and Arg-1 mRNA were measured using RT-PCR (A) and quantified by densitometry (B). The data are presented as the means±standard errors of the means (SEM); ^a^P<0.05 compared with the sham group; ^b^P<0.05 compared with the heparin+CLP group (n = 6 mices/group).

The expression of iNOS mRNA is higher in M1 macrophages. However, the expression of Arg-1 and FIZZ1 mRNAs is increased in M2 macrophages. We subjected mouse renal tissue sections to real-time PCR analysis to determine the expression of markers of M1/M2 macrophages at different time points after CLP surgery (Fig 5). Higher levels of iNOS mRNA were detected in the CLP and Ctl groups than in the heparin+CLP group. However, FIZZ1 and Arg-1 mRNA expression remained low in all three groups, indicating that HBP promoted M1 differentiation following sepsis-induced AKI.

### HBP increased TNF-α and IL-6 secretion during sepsis-induced AKI

**Fig 6:**
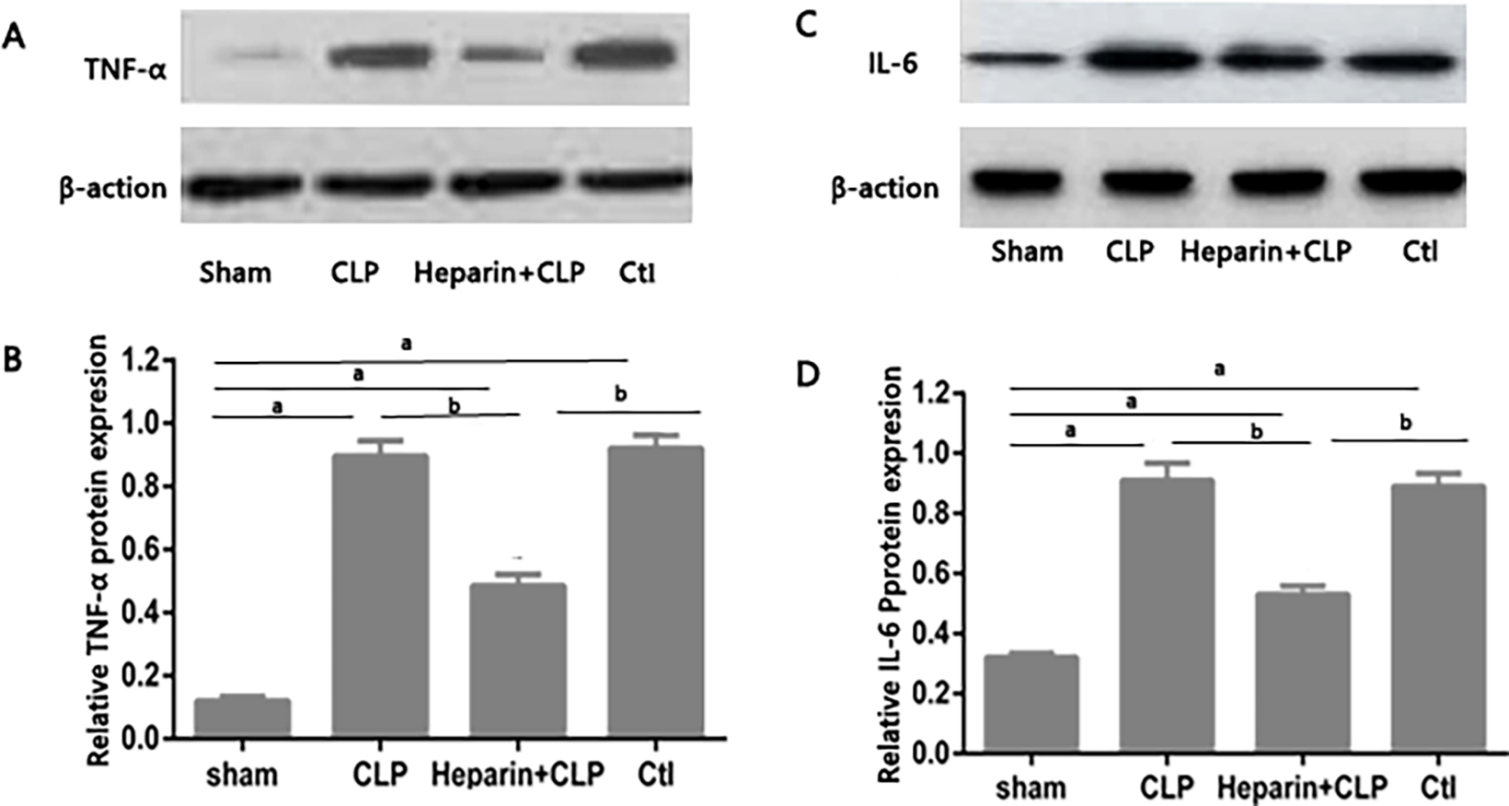
Effect of HBP on TNF-α and IL-6 protein levels with sepsis-induced AKI in mices. Levels of the TNF-α and IL-6 protein in the kidney were determined by Western blotting (A)(C) and quantified by densitometry (B)(D). Protein levels were normalized to β-actin. Graphs represent means±SEM; ^a^P<0.05 compared with the sham group; ^b^P<0.05 compared with the heparin+CLP group (n = 6 mices/group).

Because the highest total number of M1 macrophages was observed at 24 h post-surgery, we subsequently examined TNF-α and IL-6 expression at that time point (Fig 6). Western blots of whole kidney homogenates demonstrated a marked increase in total TNF-α and IL-6 protein concentrations in the CLP and Ctl groups than in the heparin+CLP group.

## Discussion

Sepsis-induced AKI is a common condition associated with high morbidity and mortality [1,16]. Macrophages are pleiotropic cells of the innate immune system, with roles spanning host defense, cytotoxicity, clearance of apoptotic cells and promotion of tissue repair. Macrophages are also known to be important mediators of renal injury in other experimental models of renal disease including transplantation, obstruction and glomerulonephritis [17]. In response to certain stimuli, macrophages can be released into the circulation from the bone marrow to migrate into target tissues and differentiate into resident macrophages. Inflammation is closely related to the activation of macrophages–M1 macrophages exhibit pro-inflammatory activities, while M2 macrophages are involved in inflammation resolution [18,19]. According to their potential mechanisms, M1 macrophages are pivotal in antigen presentation, pro-inflammatory cytokine secretion, and phagocytic activity.

The plausibility of HBP as a marker of septic organ dysfunction can be supported by its early release in response to infection and its powerful effects on immune cells and endothelial cells, which may act as causative factors in sepsis [20,21]. Heparin is a multifunctional negatively charged glycosaminoglycan that binds to HBP. Studies have suggested heparin blocks HBP-induced IL-6 production, representing a mechanism by which heparin inhibits HBP likely by blocking GAG-binding sites on HBP [22]. Evidence from recent studies has also shown that HBP activation was suppressed by heparin [23]. In this study, mice received heparin injection 12 h after the establishment of sepsis-induced AKI. HBP expression remained low after heparin injection during sepsis-induced AKI, suggesting that heparin may inhibit HBP expression.

Over the last decade, many studies have confirmed that HBP can act as a chemotactic signal for macrophages and promote the accumulation of macrophages at infected sites [24]. HBP in endothelial tissues can induce mononuclear cells to accumulate in infected sites through calcium-dependent channels. HBP can also activate mononuclear cells and increase phagocytosis of macrophages [25]. In the present study, HBP expression was particularly increased 24 h post-injury and decreased over the following hours. This is in agreement with findings that HBP expression is particularly increased during the early phase of sepsis-induced AKI when the predominant inflammatory responses occur. Our previous study has demonstrated the presence of a substantial number of M1 macrophages 24 h after sepsis-induced AKI [26]. Our findings enabled us to identify a potentially novel relation between macrophages and HBP. Thus, we can speculate that HBP may play a significant role in sepsis-induced AKI by activating M1 macrophages.

Previous studies have shown that HBP induces renal tubular cell inflammation and loss of renal endothelial cells in mice, which represent symptoms of kidney damage [27]. In this study, HBP increased renal tubular damage and renal dysfunctions and played an important role in the initial inflammatory reactions associated with renal injury. Administration of heparin after the establishment of sepsis-induced AKI reduced renal damage at 24 h after sepsis-induced AKI. Interestingly, there was also a simultaneous significant decrease in macrophage infiltration, indicating that heparin likely reduces tubular injury and macrophage infiltration by inhibiting HBP.

M1 macrophages promote the process of inflammation [6]. The main focus of our study was to investigate the potential role of HBP in the activation of M1 macrophages during sepsis-induced AKI. We sought to determine which subtype of macrophages, M1 or M2, was predominantly present in mouse renal tissue sections at 24 h after sepsis-induced AKI. Real-time PCR analysis showed increased iNOS expression in the kidneys of the four groups of mice but no increase in Arg-1 or FIZZ1 expression, suggesting that HBP activated M1 macrophages in sepsis-induced AKI.

M1 macrophages secrete high levels of pro-inflammatory cytokines (e.g., TNF-α, IL-6, IL-1β), and the generation reactive nitrogen and oxygen intermediates [28]. In the present study, we observed increased levels of TNF-α and IL-6 in the kidneys of mice subjected to CLP surgery, with less pronounced expression in the presence of heparin. Based on these results, the pro-inflammatory effects of HBP on sepsis-induced AKI is likely related to the activation of M1 macrophages.

Based on our findings, HBP plays an important role in the initial inflammatory reaction associated with sepsis-induced AKI, presumably by activating M1 macrophages and suppressing TNF-α and IL-6 secretion. Therefore, strategies that limit early macrophage infiltration or activation may represent a novel approach in the prevention or treatment of AKI in septic patients. However, the signaling pathways involved in the mechanism of activation of M1 macrophages need further investigation. Thus, our study contributes to a better understanding of the complex events involved in sepsis-induced AKI, which is key for the development of more effective therapeutic strategies.

## Supporting information

S1 Dataset Dataset showing the data for Fig 3C (Scr) and 3D (BUN). (XLSX)

S1 Fig HBP mRNA levels following sepsis-induced AKI in mices. Levels of the HBP mRNA were measured using RT-PCR (0, 6, 12, 24, 36 and 48h).
(TIF)

S2 Fig HBP mRNA levels following sepsis-induced AKI in mices. Levels of the HBP mRNA were measured using Western blotting (0, 6, 12, 24, 36 and 48h).
(TIF)

S3 Fig Twenty-four hours prior to the onset of CLP, mices were treated with heparin. Levels of the HBP protein in the kidney were determined by Western blotting.
(TIF)

S4 Fig Photomicrographs of H&E-stained kidney sections (200x). (TIF)

S5 Fig Expression of the CD68 protein was detected by immunohistochemical staining (200x). (TIF)

S6 Fig Levels of the iNOS and Arg-1 mRNA were measured using RT-PCR. (TIF)

S7 Fig Levels of the TNF-α protein in the kidney were determined by Western blotting. (TIF)

S8 Fig Levels of the IL-6 protein in the kidney were determined by Western blotting. (TIF)
