# Supplementary figures and images for: Activation of M1 macrophages in sepsis-induced acute kidney injury in response to heparin-binding protein
Source: PLoS One. 2018 May 3;13(5):e0196423. doi: 10.1371/journal.pone.0196423 (PMC5933766; doi:10.1371/journal.pone.0196423)

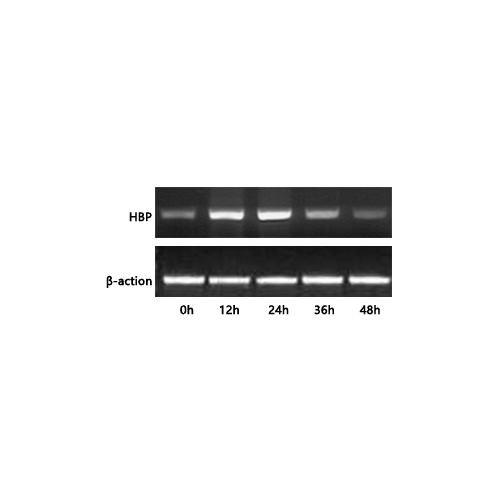

Supplement: S1 Fig — Levels of the HBP mRNA were measured using RT-PCR (0, 6, 12, 24, 36 and 48h). (TIF) [file pone.0196423.s002.tif]

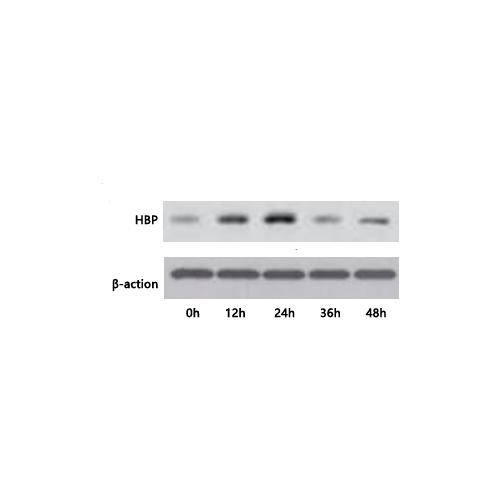

Supplement: S2 Fig — Levels of the HBP mRNA were measured using Western blotting (0, 6, 12, 24, 36 and 48h). (TIF) [file pone.0196423.s003.tif]

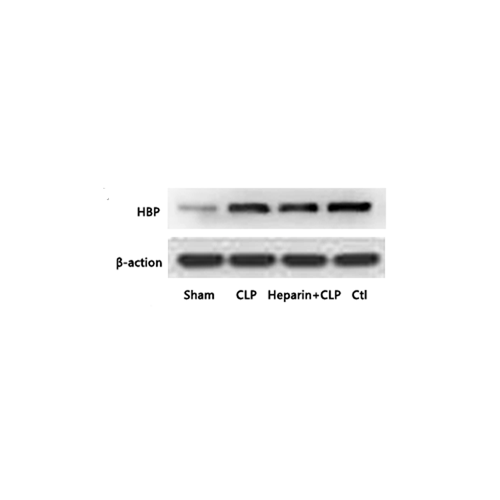

Supplement: S3 Fig — Levels of the HBP protein in the kidney were determined by Western blotting. (TIF) [file pone.0196423.s004.tif]

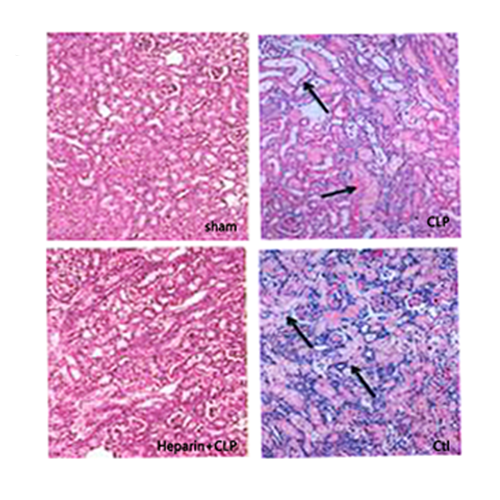

Supplement: S4 Fig — (TIF) [file pone.0196423.s005.tif]

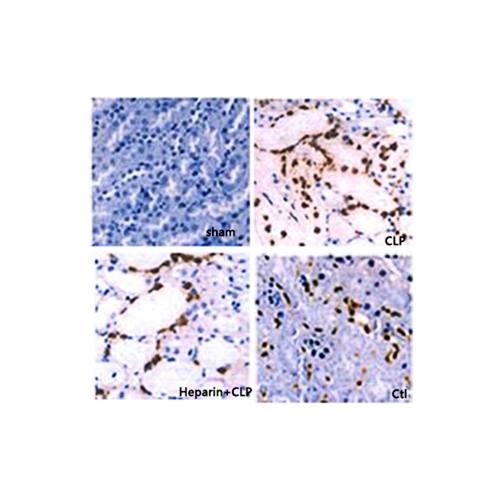

Supplement: S5 Fig — (TIF) [file pone.0196423.s006.tif]

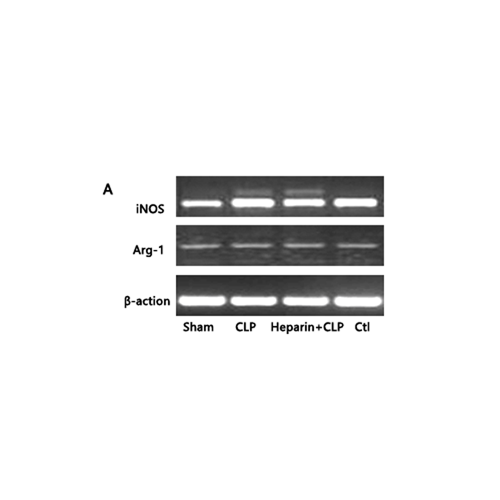

Supplement: S6 Fig — (TIF) [file pone.0196423.s007.tif]

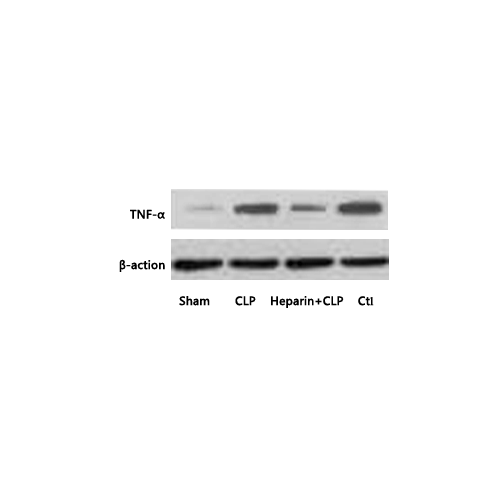

Supplement: S7 Fig — (TIF) [file pone.0196423.s008.tif]

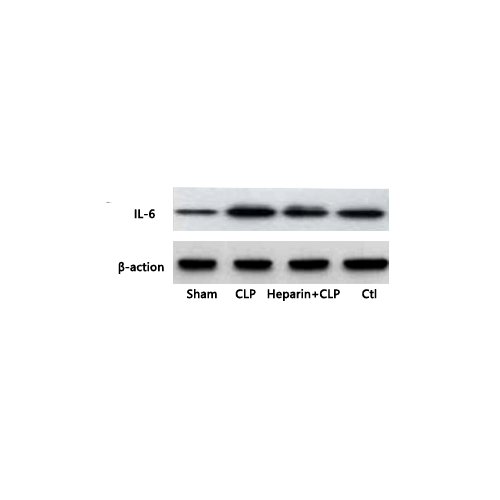

Supplement: S8 Fig — (TIF) [file pone.0196423.s009.tif]
